# Neoadjuvant Chemotherapy Followed by Radical Surgery versus Radiotherapy (with or without Chemotherapy) in Patients with Stage IB2, IIA, or IIB Cervical Cancer: A Systematic Review and Meta-Analysis

**DOI:** 10.1155/2020/7415056

**Published:** 2020-07-27

**Authors:** Qingjian Ye, Yuebo Yang, Xinran Tang, Jing Li, Xiaomao Li, Yu Zhang

**Affiliations:** Department of Gynecology, The Third Affiliated Hospital of Sun Yat-sen University, Guangzhou 510630, China

## Abstract

**Background:**

This study was to compare the efficacy and safety between neoadjuvant chemotherapy followed by radical surgery (NACT+RS) and radiotherapy only (RT) or concurrent chemoradiotherapy (CCRT) for treatment of patients with stage IB2, IIA, or IIB cervical cancer.

**Method:**

The electronic databases of PubMed, Embase, and the Cochrane Library were searched to screen relevant studies from their inception to October 2018. Clinical data including overall survival (OS), disease-free survival (DFS), and adverse events were extracted. Egger's test was used to evaluate the publication bias, and sensitivity analysis was conducted to estimate the robustness of results.

**Results:**

Finally, three randomized controlled trials (RCTs) and two case-control studies consisting of 1,275 patients with stage IB2, IIA, or IIB cervical cancer were included in the current study. Overall, pooled results showed no significant differences in OS ((hazard ratio (HR) = 0.603, 95%CI = 0.350 − 1.038) and DFS (HR = 0.678, 95%CI = 0.242 − 1.904) for patients treated with NACT+RS compared with RT only or CCRT, but the subgroup analysis showed that the OS and DFS were significantly longer in the NACT+RS groups than the RT or CCRT group (OS: HR = 0.431, 95%CI = 0.238 − 0.781, *p* = 0.006; DFS: HR = 0.300, 95%CI = 0.187 − 0.482, *p* < 0.001) for the population with median follow-up time of more than 60 months. For adverse events, the incidence of thrombocytopenia in the NACT+RS group was significantly higher than that in the RT only or CCRT group (relative risk (RR) = 3.240, 95% CI 1.575-6.662), while the incidence of diarrhea was significantly lower than that in the RT only or CCRT group (RR = 0.452, 95% CI =0.230-0.890).

**Conclusion:**

These findings suggest that the short-term therapeutic effects of the two treatments may be possibly equal for patients with stage IB2-IIB cervical cancer, but the long-term effects for improving OS and DFS may be better using NACT+RS compared with the RT only or CCRT.

## 1. Introduction

Cervical cancer is the second most common malignant tumor in women and has become a major public health problem worldwide [1, 2]. In developing countries, more than 80% of patients with cervical cancer are diagnosed at an advanced stage, which seriously affects the prognosis of the sufferers [3]. Locally advanced cervical cancer (LACC) is defined as IB2-IIB stages according to the International Federation of Gynecology and Obstetrics (FIGO) staging system [4], treatment of which may reduce the risk of progression and subsequent death [5, 6]. Since 2000, concurrent chemoradiotherapy (CCRT) is recommended as the standard treatment for LACC in some countries [7, 8], which has been demonstrated to prolong the survival by approximately 50% compared with radiotherapy (RT) alone [5, 6]. However, a meta-analysis revealed that there were still 25% to 40% of patients with LACC experiencing relapse and the time to metastases at 5 years was also not significantly improved after CCRT compared with RT [9], implying the necessity to explore more effective therapeutic strategies.

Neoadjuvant chemotherapy (NACT) followed by radical surgery (RS) has been extensively explored over the past decade as it could reduce the requirement of postoperative radiotherapy and thus improve the prognosis [10, 11]. NACT can shrink tumor size and hence transform inoperable into radically resected tumors [12]. Also, via a systematic review and meta-analysis of six randomized controlled trials (RCTs) (1,072 women), Rydzewska et al. found that the combination of NACT and RS was superior to surgery alone in improving progression-free survival (hazard ratio (HR) = 0.76, 95%confidence intervals (CI) = 0.62 to 0.94, *p* = 0.01] for LACC patients [13]. A meta-analysis of five RCTs performed by Peng et al. also suggested that NACT+RS could reduce the risk of lymph node metastasis, interstitial infiltration, vascular infiltration, and positive incision margin compared to the RS-alone group, which were all prognostic-related factors [14]. These findings indicated that NACT+RS may be an attractive alternative treatment option for LACC, in addition to CCRT or RT.

However, whether the therapeutic effect of NACT+RS is better than RT alone or CCRT remains unclear because controversial conclusions were reported by previous studies. For example, Gupta et al. observed that the 5-year disease-free survival (DFS) in the NACT+RS group was lower than that of the CCRT group (69.3% vs. 76.7%, *p* = 0.038), whereas the corresponding 5-year overall survival (OS) was significant different between two groups [15]. Yin et al. proved that the NACT+RS group had significantly higher survival rates than the CCRT group (DFS: HR = 3.535, *p* < 0.0001; OS: HR = 3.157, *p* < 0.0001) [16]. These differences may be attributed to a small sample size of an individual study. Therefore, it is indispensable to comprehensively evaluate the effects of these therapeutic strategies for patients with LACC in the current study using a systematic review and meta-analysis.

## 2. Methods

### 2.1. Search Strategy

The electronic databases of PubMed, Embase, and Cochrane were systematically searched to screen the relevant studies up to October 2018. In addition, the references included in the searched literatures and related reviews were also searched to prevent eligible articles from being omitted. The search terms included (1) “cervical cancer” or “uterine cancer”; and (2) “neo-adjuvant chemotherapy” or “preoperative chemotherapy”; and (3) “radiotherapy” or “chemoradiotherapy”. The language was not limited when literature searching was performed.

### 2.2. Inclusion and Exclusion Criteria

Studies that met the following criteria were included in this study. (1) Subjects were cervical cancer patients with stage IB2-IIB. (2) Patients in the study group underwent NACT+RS. (3) Patients in the control group underwent RT only or CCRT. (4) OS, DFS, or both were used as the study endpoint. (5) DFS- or OS-related data can be extracted. Detailed exclusion criteria were listed as follows. (1) Study subjects were noncervical cancer. (2) Studies were descriptive such as case reports, reviews, and guidelines. (3) Study endpoint was not about survival time. (4) Studies did not compare the effects of NACT+RS with RT or CCRT. (5) Study subjects were not patients with stage IB2-IIB cervical cancer. Two researchers independently searched and screened the literatures. If there was an inconsistency between the two researchers, a third investigator was enrolled to resolve it.

### 2.3. Data Extraction and Quality Assessment

Data extraction was also independently carried out by two researchers based on preestablished forms. Considering that patients with cervical cancer of stage III or more serious stages are not suitable for surgery, this study only compared the efficacy of patients with IB2-IIB cervical cancer. OS and DFS were taken as the primary objective, and the adverse events were used as secondary research results. Basic characteristics (including author, publication year, country, design, FIGO stage, NACT method, and follow-up time from studies that met the inclusion criteria) were extracted. If the OS or DFS data in the study could not be extracted directly, Engauge Digitizer 4.1 was used to extract the corresponding data through Kaplan-Meier survival curves. Adverse events in this study mainly included anemia, thrombocytopenia, neutropenia, leukopenia, vomiting, diarrhea, and neurotoxicity. The quality of the included studies in this meta-analysis was evaluated according to the guidelines in the Cochrane Handbook for Systematic Reviews of Interventions in Chapter 8 [17]. This study was approved by the ethics committee of the Third Affiliated Hospital of Sun Yat-sen University.

### 2.4. Statistical Analysis

Stata statistical software (version 14.0, Stata Corporation, College Station, TX, USA) was used for statistical analysis and Review Manager (version 5.3.3, Cochrane Collaboration, Oxford, UK) was used for quality evaluation of literatures. Summary HR and corresponding 95% CI were calculated from pooled data for OS and DFS, while the risk ratio (RR) value and the corresponding 95% CI were computed to compare adverse events between the NACT+RS group and the RT or CCRT group. Cochran's *Q* test and *I*^2^ statistic were involved to quantify the heterogeneity of the included studies. If the *I*^2^ value was greater than 50%, the heterogeneity was considered to be obvious and thus the random-effects model was used for the meta-analysis; otherwise, a fixed effects model was applied. Egger's test was used to assess the publication bias. A sensitivity analysis was performed by using the “remove one study” method to evaluate the impact of individual studies on overall results.

## 3. Results

### 3.1. Study Selection and Quality Assessment

**Figure 1:**
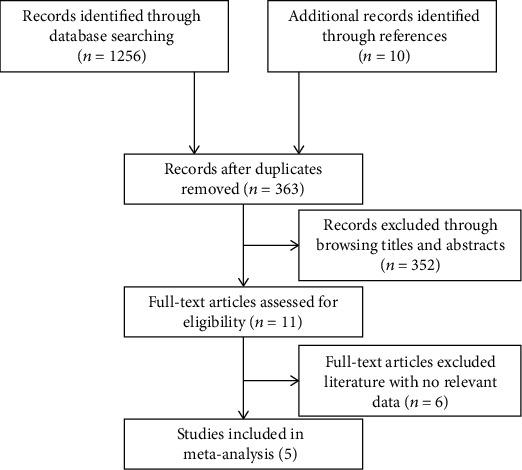
Flow diagram of the entire research literature.

Detailed literature search and screening processes are shown in Figure 1. A total of 1,266 related articles were retrieved, including 1,256 directly obtained through searching PubMed, Embase, and Cochrane Library databases and 10 articles after reading the references or related reviews. Of these, 903 studies were removed due to duplicate literatures. Among the left 363 articles, 358 articles were further excluded because they were case reports (*n* = 29), 134 articles of noncervical cancers (*n* = 134), descriptive studies (*n* = 26), unrelated topics (*n* = 114), systematic reviews and meta-analyses (*n* = 8), neoadjuvant chemotherapy and no surgery (*n* = 7), nonprognosis (*n* = 18), early cervical cancer (*n* = 7), and without neoadjuvant (*n* = 9). Finally, five articles that met the inclusion criteria were enrolled in the current study.

### 3.2. Study Characteristics and Quality Assessment

**Figure 2:**
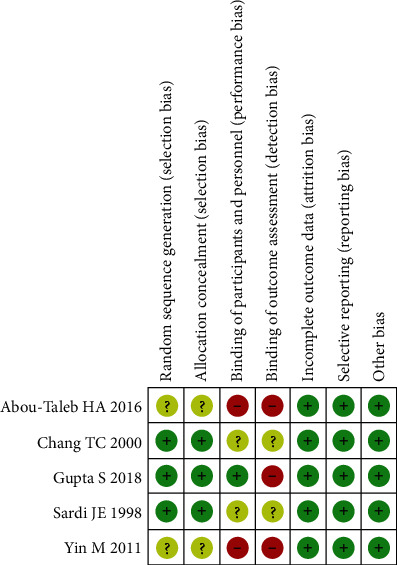
Risk of bias summary for the five included studies.

These five studies included two case-control studies and three RCTs and were published from 1998 to 2018 [15, 16, 18–20]. The main characteristics of the studies are listed in Table 1. A total of 1,275 patients with stage IB2-IIB cervical cancer were involved in this study. In order to identify the quality of the included studies, the Cochrane Collaboration risk of bias tool was used. Two studies did not describe the information of random sequence generation and the allocation concealment, and three studies did not use a blinded mode to assess the outcome. The detailed quality evaluation results are shown in Figure 2.

### 3.3. Pooled Analysis for OS

**Figure 3:**
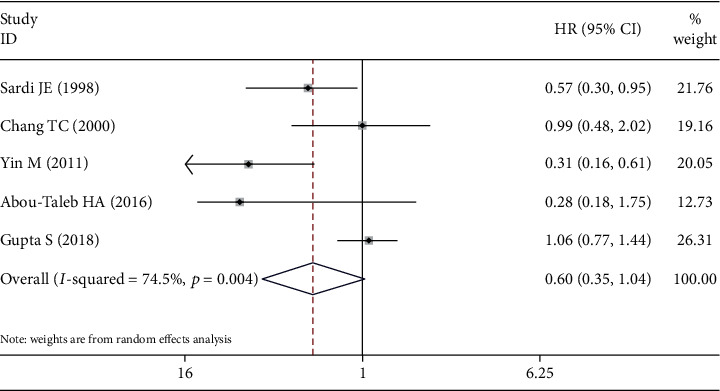
Forest plots for the overall survival.

All of the five studies compared the OS outcome of NACT+RS with RT or CCRT for cervical cancer patients with stage IB2-IIB [15, 16, 18–20]. A random effects model was chosen for meta-analysis because of the large degree of heterogeneity between studies (*I*^2^ = 74.5%, *p* = 0.004). The pooled results showed that compared to RT or CCRT, NACT+RS did not significantly extend the OS of patients with stage IB2-IIB cervical cancer (HR = 0.603, 95%CI = 0.350 − 1.038; p = 0.068) (Figure 3).

### 3.4. Pooled Analysis for DFS

Four of the included studies reported DFS in 1,034 patients with stage IB2-IIB cervical cancer, with a median follow-up of 39.0-82.8 months [15, 16, 19]. Similar to OS, there was a large heterogeneity in DFS; therefore, a random effects model (*I*^2^ = 93.5%, *p* < 0.001) was performed for summary analysis. The summary results also showed no difference in DFS between the NACT+RS group and the RT or CCRT group, indicating that NACT+RS could not increase DFS (HR = 0.678, 95%CI = 0.242 − 1.904; *p* < 0.001) (Supplemental figure 1).

### 3.5. Subgroup Analysis

In order to explore the source of heterogeneity among studies, a subgroup meta-analysis was conducted according to country, publication year, sample size, follow-up time, study design, and control therapy. As illustrated in Table 2, the OS and DFS of patients who undertook NACT+RS were found to be significantly longer than that of the RT or CCRT group (OS: HR = 0.431, 95%CI = 0.238 − 0.781, *p* = 0.006; DFS: HR = 0.300, 95%CI = 0.187 − 0.482, *p* < 0.001) in the population with median follow-up time of more than 60 months, indicating the NACT+RS may be more effective than the RT or CCRT group for improving the long-term survival for cervical cancer patients with stage IB2-IIB. The same situation was identified in studies designed as case-control trials (Table 2: OS: HR = 0.302, 95%CI = 0.170 − 0.538, *p* < 0.001; Table 3: DFS: HR = 0.300, 95%CI = 0.187 − 0.482, *p* < 0.001).

### 3.6. Adverse Events

In order to compare the safety of the two treatment methods, a pooled analysis of the adverse events was undertaken. No significant differences in anemia, neutropenia, leukopenia, vomiting, or neurotoxicity was observed between the two groups (Supplemental table 1). However, the incidence of thrombocytopenia was significantly higher in the NACT+RS group than in the RT or CCRT group (RR = 3.240, 95%CI = 1.575 − 6.662, *p* = 0.001), while the RT or CCRT group was more prone to develop diarrhea than the NACT+RS group (RR = 0.452, 95%CI = 0.230 − 0.890, *p* = 0.022).

### 3.7. Publication Bias and Sensitivity Analysis

Potential publication bias of the included studies was investigated using the Egger test. As a result, no obvious publication bias was found for OS and DFS (OS: *p* = 0.131; DFS: *p* = 0.461). Additionally, a sensitivity analysis was performed to investigate the impact of individual study on the results of the pooled analysis. The result showed that a single study had little effect on the summary results, which indicated that the results of this study were robust (Supplemental figure 2).

## 4. Discussion

This meta-analysis compared the efficacy of NACT+RS with RT or CCRT for patients with stage IB2-IIB cervical cancer by summarizing the results of five studies. The results suggested no differences in OS and DFS between the two groups. These results seemed to be inconsistent with the study of Tierney who also used five studies to conduct a pooled analysis to compare the effects of NACT+RS with RT only [11] and the results showed that NACT+RS could prolong the OS and DFS compared with RT only in patients with cervical cancer. These differences may be attributed to the fact that stage III patients were excluded in our study as stage III patients are usually not allowed to undergo surgery. It is worth noting that for LACC, the US standard treatment is CCRT [5, 6, 21, 22], while some institutions in Europe and Asia are NACT+RS [23–25]. In subgroup analysis, the impact of NACT+RS versus CCRT on survival was also compared. Similarly, no differences in OS and DFS were identified between patients using these two treatments. However, we found that the OS and DFS of the NACT+RS group were longer compared with the CCRT group for patients with a median follow-up of more than 60 months, indicating the long-term effects of NACT+RS may be more superior. But, it should be noted that the studies included in this meta-analysis was not large enough, and more multicenter studies with longer follow-up are needed to verify this conclusion.

Adverse events, which are highly linked to a specific situation (whether the course of treatment can be completed on schedule), are one of the issues that must be considered during treatment. According to the report of Abou-Taleb et al., patients treated with irinotecan/nedaplatin for NACT are less likely to suffer from severe anemia and vomiting than those receiving CCRT [18]. Our study did not find that the neoadjuvant therapy could reduce the incidence of severe anemia and vomiting compared with the RT or CCRT group in patients with stage IB2-IIB cervical cancer. Pignata and coworkers argued that diarrhea was the only severe and dose-limiting nonhematologic toxicity associated with CCRT [26]. This study demonstrated that the incidence of diarrhea in the CCRT or RT group was higher than that in the NACT+RS group, which was consistent with that of Pignata et al. [26].

Our meta-analysis had many advantages. This was the first study to evaluate the efficacy and safety of NACT+RS versus RT only or concurrent CCRT in patients with stage IB2, IIA, or IIB cervical cancer. Because cervical cancer patients with stage IB2-IIB are often available for surgery, this evaluation is particularly clinically instructive. In addition, we conducted quality assessments, publication biases, and sensitivity analyses on the included studies, which ensured the reliability of the results. At the same time, this study had some limitations. First, only five articles were included in this meta-analysis; therefore, the number of documents was relatively small. Second, this study only compared the survival rate and adverse events but did not compare the quality of life due to the lack of relevant report data. The quality of life is also a key factor in the choice of treatment. In 2013, Le Borgne and colleagues studied the long-term health-related quality of life in cervical cancer and found that quality of life of survivors receiving radiation therapy was significantly worse in terms of sexual dysfunction, urination and abdominal symptoms, and lymphedema compared with survivors who only received surgery [27].

In conclusion, this systematic review and meta-analysis suggests that the short-term therapeutic effects of the two treatments may be possibly equal for patients with stage IB2-IIB cervical cancer, but the long-term effects on OS and DFS may be better using NACT+RS compared with the RT only or CCRT. NACT+RS may be especially applicable in some areas where radiotherapy equipment is in short supply. This meta-analysis provides the basis for the patients with cervical cancer to choose the better treatment plan and calls for the increase of radiotherapy equipment.

## Figures and Tables

**Table 1:** The main characteristics of the five included studies.

Study	Year	Country	Design	Case group		Control group			FIGO stage	NACT	Follow-up (month)	Primary endpoints
				*n*	Age	*n*	Age	Therapy				
Gupta [16]	2018	India	RCT	316	50	317	48	CCRT	IB2-IIB	3 cycles of paclitaxel and carboplatin	58.5	OS,DFS
Abou-Taleb [17]	2016	Japan	Case control	70	53	22	77	RT	IB2-IIB	1-3 cycles of irinotecan and nedaplatin^&^	Unclear	OS
Yin [18]	2011	China	Case control	187	43	94	47	CCRT	IB2-IIB	2 or 3 cycles of cisplatin	82.8	OS,DFS
Chang [19]	2000	Taiwan	RCT	68	46	52	47	RT	IB2-IIA	3 cycles of VBP scheme^#^	39.0	OS,DFS
Sardi [20]	1998	Argentina	RCT	76	44^∗^	73	41.5^∗^	RT	IIB	3 cycles of VBP scheme	84.0	OS

Abbreviations: FIGO: International Federation of Gynecology and Obstetrics; NACT: neoadjuvant chemotherapy; RS: radical surgery; CCRT: concurrent chemoradiotherapy; RT: radiotherapy; OS: overall survival; DFS: disease-free survival; ^∗^mean age. Age values are presented as median; follow-up time is presented as median. ^&^NACT was performed using transuterine arterial chemotherapy (TUAC) or intravenous. ^#^The VBP scheme was used (3 courses of vincristine 1 mg/m^2^ on day 1, bleomycin 25 mg/m^2^ on days 1 to 3, and cisplatinum 50 mg/m^2^ on day 1, at 10-day intervals).

**Table 2:** The results of pooled HR for OS in different subgroups.

	No.	HR	95% CI	*P*	*I*-squared	*p*
Country						
Asian	4	0.598	0.295-1.212	0.154	79.2%	0.004
Other	1	0.570	0.320-1.014	0.056	—	—
Publication year						
Before 2010	2	0.719	0.422-1.227	0.227	27.5%	0.240
After 2010	3	0.488	0.179-1.325	0.159	85.8%	0.001
Sample size						
<150	3	0.602	0.332-1.092	0.095	19.7%	0.166
>150	2	0.595	0.179-1.980	0.397	90.6%	0.001
Follow-up time						
≤60	2	1.049	0.787-1.397	0.746	0.0%	0.864
>60	2	0.431	0.238-0.781	0.006	45.3%	0.176
Unclear	1	0.280	0.090-0.873	0.028	—	—
Study design						
RCT	3	0.877	0.595-1.291	0.505	42.4%	0.176
Case control	2	0.302	0.170-0.538	0.000	0.0%	0.880
Controls therapy						
CCRT	2	0.595	0.179-1.980	0.397	90.6%	0.001
RT	3	0.603	0.332-1.092	0.095	44.2%	0.166

Abbreviations: CCRT: concurrent chemoradiotherapy; RT: radiotherapy.

**Table 3:** The results of pooled HR for DFS in different subgroups.

	No.	HR	95% CI	*P*	*I*-squared	*p*
Country						
Asian	3	0.678	0.242-1.904	0.461	93.5%	0.000
Other	0	—	—	—	—	—
Publication year						
Before 2010	1	0.690	0.370-1.288	0.244	—	—
After 2010	2	0.669	0.142-3.154	0.611	96.7%	0.000
Sample size						
<150	1	0.690	0.370-1.288	0.244	—	—
>150	2	0.669	0.142-3.154	0.611	96.7%	0.000
Follow-up time						
≤60	2	1.057	0.511-2.187	0.882	77.6%	0.035
>60	1	0.300	0.187-0.482	0.000	—	—
Study design						
RCT	2	1.057	0.511-2.187	0.882	77.6%	0.035
Case control	1	0.300	0.187-0.482	0.000	—	—
Control therapy						
CCRT	2	0.669	0.142-3.154	0.611	96.7%	0.000
RT	1	0.690	0.370-1.288	0.244	—	—

Abbreviations: CCRT: concurrent chemoradiotherapy; RT: radiotherapy.

## Data Availability

The data supporting this meta-analysis are from previously reported studies and datasets, which have been cited.

## Supplementary Materials

Supplementary Materials Supplementary 1 Supplemental Figure 1 Forest plots for the DFS. Supplementary 2 Supplemental Figure 2 Publication bias and sensitivity analysis for OS and DFS. (A) Publication bias for overall survival. (B) Publication bias for disease-free survival. (C) Sensitivity analysis for overall survival. (D) Sensitivity analysis for disease-free survival.
