# Supplementary material for: Neoadjuvant Chemotherapy Followed by Radical Surgery versus Radiotherapy (with or without Chemotherapy) in Patients with Stage IB2, IIA, or IIB Cervical Cancer: A Systematic Review and Meta-Analysis
Source: Dis Markers. 2020 Jul 27;2020:7415056. doi: 10.1155/2020/7415056 (PMC7403931; doi:10.1155/2020/7415056)

Supplemental figure 1

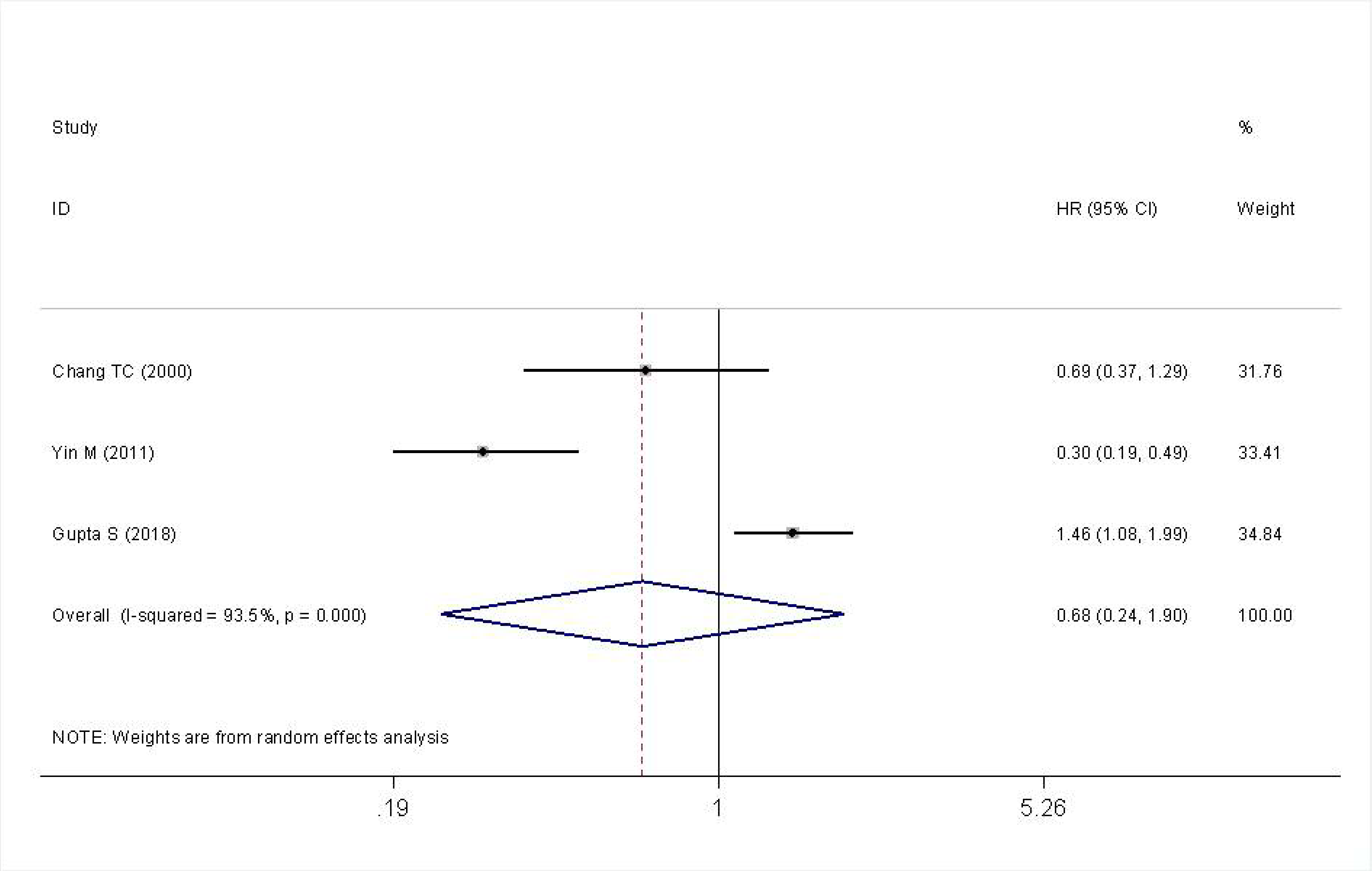

Supplemental table 1. Comparison of adverse events between NACT+RS and CCRT or RT alone for patients with locally advanced cervical cancer

Adverse events	NO.	RR	95%CI	P	I-squared	p	model
Anemia	3	0.759	0.202-2.845	0.683	87.4%	<0.001	random
Thrombocytopenia	3	3.240	1.575-6.662	0.001	0.0%	0.782	fixed
Neutropenia	2	1.323	0.891-1.964	0.165	0.0%	0.388	fixed
Leukopenia	2	2.263	0.068-75.166	0.648	90.4%	0.001	random
Vomiting	4	0.991	0.292-3.357	0.988	82.1%	0.001	random
Diarrhea	4	0.452	0.230-0.890	0.022	77.6%	0.002	random
Neurotoxicity	2	0.292	0.074-1.146	0.078	52.6%	0.146	fixed

Supplemental figure 2

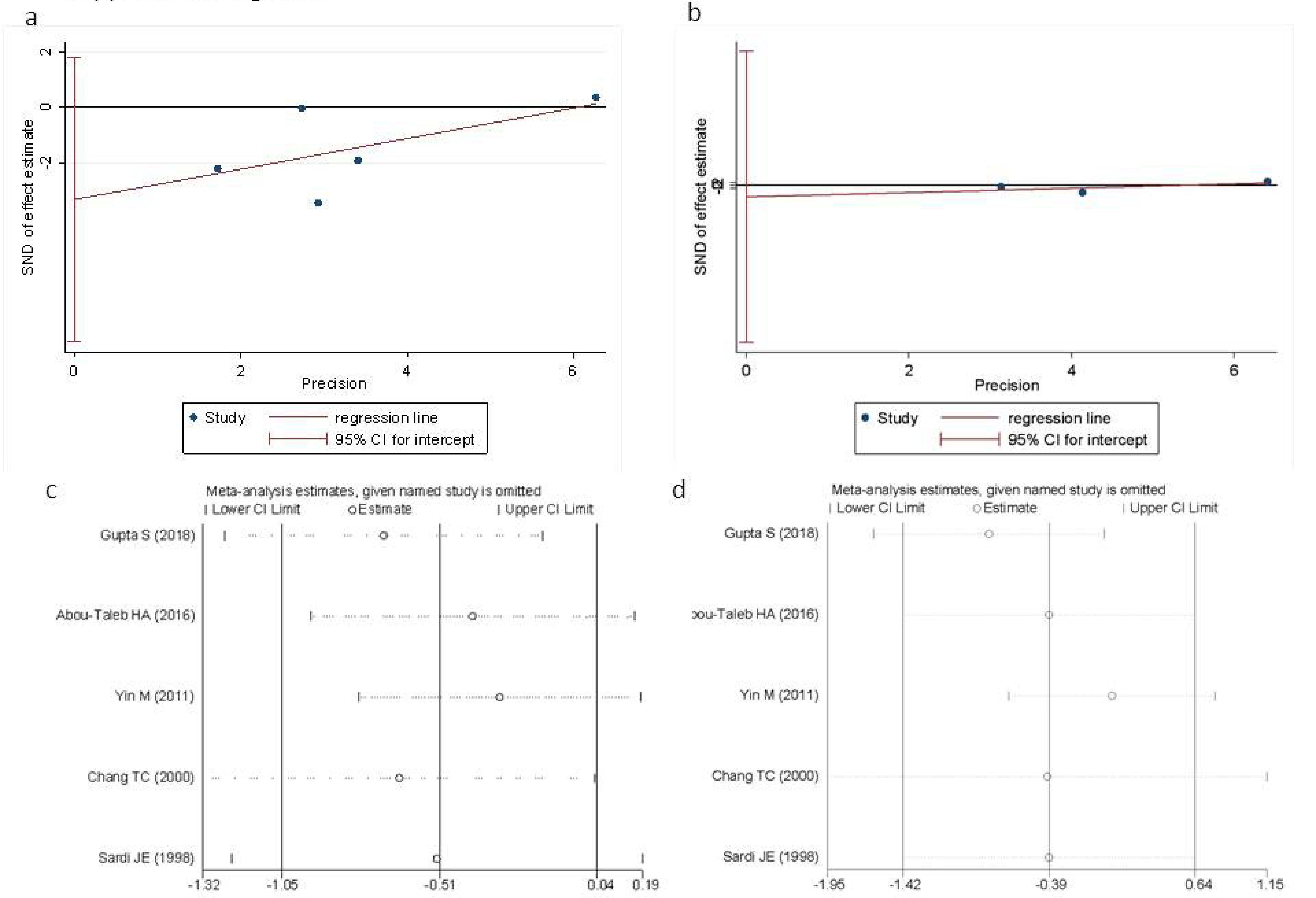

Supplement: Supplementary Materials — Supplementary 1 Supplemental Figure 1 Forest plots for the DFS. Supplementary 2 Supplemental Figure 2 Publication bias and sensitivity analysis for OS and DFS. (A) Publication bias for overall survival. (B) Publication bias for disease-free survival. (C) Sensitivity analysis for overall survival. (D) Sensitivity analysis for disease-free survival. [file 7415056.f1.pdf]
